# Aberrant Hypermethylation of Aldehyde Dehydrogenase 2 Promoter Upstream Sequence in Rats with Experimental Myocardial Infarction

**DOI:** 10.1155/2015/503692

**Published:** 2015-01-05

**Authors:** Peng Wang, Cheng Shen, Lei Diao, Zhiyin Yang, Fan Fan, Cong Wang, Xiangwei Liu, Xiaolei Sun, Zhen Dong, Hong Zhu, Xin Ma, Quan Cao, Xiaona Zhao, Duan Ma, Yunzeng Zou, Kai Hu, Aijun Sun, Junbo Ge

**Affiliations:** ^1^Shanghai Institute of Cardiovascular Diseases, Zhongshan Hospital, Fudan University, Shanghai 200032, China; ^2^Department of Cardiology, Shandong University, Jinan, Shandong 250012, China; ^3^Institutes of Biomedical Sciences, Fudan University, Shanghai 200032, China; ^4^Department of Cardiology, Jining Medical College, Jining, Shandong 272067, China

## Abstract

*Background*. Aldehyde dehydrogenase 2 (ALDH2) plays a crucial role in myocardial protection against ischemia. Downregulation of ALDH2 was evidenced after myocardial infarction and the underlying mechanism is not fully understood. DNA methylation can regulate gene transcription in epigenetic level. We thus hypothesized that DNA methylation may affect ALDH2 expression in myocardial infarction (MI). *Methods*. MI was induced in Sprague-Dawley rats. MI border zone tissues were harvested at 1st week, 2nd week, and 3rd week after MI. Bisulfite sequencing PCR (BSP) was performed to detect the methylation levels of ALDH2 core promoter. Sequenom MassARRAY platform (MassARRAY) was used to examine the methylation levels of ALDH2 promoter upstream sequence. ALDH2 protein and mRNA expression were assayed by Western blot and real-time PCR, respectively. *Results*. Compared with Sham group, ALDH2 protein and mRNA expression of MI groups was significantly downregulated. Compared with Sham group, DNA methylation level of CpG sites in ALDH2 promoter upstream sequence was significantly higher in MI groups in a time-dependent manner (CpG1, CpG2, and CpG7, *P* < 0.01). DNA methylation did not affect ALDH2 core promoter sequence during the progress of MI. No significant difference was detected in DNA methylation level of ALDH2 promoter upstream sequence among MI groups. *Conclusion*. Aberrant hypermethylation of CpG sites in ALDH2 promoter upstream sequence is associated with myocardial ischemia injury and may partly result in ALDH2 downregulation after MI.

## 1. Introduction

Previous study shows aldehyde dehydrogenase 2 (ALDH2) plays a critical role in myocardial protection against ischemia, and down-regulation of ALDH2 expression is associated with exacerbated myocardial ischemia injury [1]. Furthermore, enhanced activation of ALDH2 could ameliorate myocardial ischemia injury in rats with experiment myocardial infarction (MI) [2]. The underlying mechanisms for the protective effects of ALDH2 against ischemia are multiple, such as decreasing toxic aldehydes accumulation [1], enhancing autophagy via AMPK-and Akt/mTOR signal [3], promoting apoptosis by MAPK-ERK1/2-JNK-p38 pathway [4] and modulating ER stress [5]. Our previous study demonstrated ALDH2 of myocardial cells decreased after MI, both in transcription and translation level [6]. However, the underlying mechanisms of ALDH2 reduction post MI remain largely unknown, moreover, there is no study focusing on upstream regulation of ALDH2 at transcription level.

In the light of the epigenetic theory [7], DNA methylation can regulate gene transcription by adding methyl groups to cytosine residues into DNA sequences [8–10]. DNA methylation can induce repression of gene transcription, physically preventing transcription factor binding and reducing access to gene regulatory regions [11, 12]. There are reports indicating DNA methylation involvement in myocardial protection against ischemia by affecting the transcription level of specific genes [13]; we, hence, hypothesized that DNA methylation level of ALDH2 gene may affect its transcription or be involved in myocardial protection in the setting of MI.

## 2. Methods

### 2.1. MI Model and Study Groups

Adult male Sprague-Dawley rats weighing 240–250 g were purchased from Shanghai Animal Administration Center. MI was produced by left anterior descending (LAD) artery ligation as described previously [14]. Success of MI was proved by echocardiography on the 7th day after surgery. Animal experimental protocols were operated on according to the guidelines of “The Guide for the Care and Use of Laboratory Animals” and were approved by the Animal Care and Use Committee of Fudan University Academy Press (NIH Publication number 85-23, revised 1996).

Experimental rats were randomized into 4 groups, which included one Sham group and three MI experiment groups. Each MI group contained 5 successfully LAD ligations with reduced ejection fraction (<40%) and myocardial tissue was harvested at 1 week, 2 weeks, and 3 weeks after MI, respectively.

### 2.2. DNA Extraction

Genomic DNA was extracted from the infarction border using a QIA amp DNA Mini Kit according to manufacturer's instructions (Qiagen, Hilden, Germany), and myocardial tissue in the same region of Sham group was also obtained for DNA extraction. The concentration and purity of the DNA were determined by absorbances at 260 and 280 nm by NanoDropTM 1000 spectrophotometer (Thermo Scientific, Wilmington, USA).

### 2.3. DNA Sodium Bisulfite Conversion

Sodium bisulfite modification for the extracted DNA was performed using an EZ DNA Methylation Kit^*TM*^ according to manufacturer's instructions (Zymo Research, Orange, CA, USA). Sequencing results confirmed that more than 99.0% of cytosine residues were converted. The bisulfite-converted DNA was resuspended in 10 *μ*L elution buffer and stored at −80°C for BSP and MassARRAY.

### 2.4. Bisulfite Sequencing PCR for ALDH2 Core Promoter

CpG and CpG islands of ALDH2 core promoter BSP were determined with online software (http://emboss.bioinformatics.nl/cgi-bin/emboss/cpgplot); primers of ALDH2 core promoter were designed with Primer software Methyl Primer Express v1.0.exe and the primers were shown in Table 1.

A 20 *μ*L mixture was prepared for each reaction and included 1x HotStarTaq buffer, 2.0 mM Mg2+, 0.2 mM dNTP, 0.2 *μ*M of each primer, 1U HotStarTaq polymerase (Qiagen Inc.), and 1 *μ*L template DNA. The cycling program was 95°C for 15 min: 11 cycles of 94°C for 20 s, 62°C–0.5°C per cycle for 40 s, and 72°C for 1 min; 24 cycles of 94°C for 20 s, 56°C for 30 s, and 72°C for 1 min, and then 72°C for 2 min. PCR products were purified by adding 1U SAP and 6U Exo I per 8U PCR products. The mixtures were incubated at 37°C for 60 min, followed by incubation at 70°C for 10 mins.

Sequencing reaction were performed in reaction mixture including 2 *μ*L BigDye3.1 mix, 2 *μ*L sequencing primer (0.4 *μ*M), and 1-2 *μ*L purified PCR product, and sequencing primers are GTGAGTTGGGTAGGGATGGA(ALDH2-MF1), GGGATAAAGAGGATTGTTTAGGATA(ALDH2-MF2), CCRTATCTCTACCTCCCATTAATAACC(ALDH2-MF3), and ATCCCCATATTCTACAAACTCCAT CTC(ALDH2-MF4). All of sequencing primers were designed with MethPrimer software. The cycling program was 96°C for 1 min: 28 cycles of 96°C for 10 s, 50°C for 5 s, and 60°C for 4 min.

Final results were evaluated in ABI3130XL sequencer.

### 2.5. MassARRAY Quantitative Methylation Analysis

The Sequenom MassARRAY platform was used for the quantitative methylation analysis of the upstream sequence of ALDH2 gene promoter. The methylation status of a detected pattern was then analyzed using Epityper software version 1.0 (Sequenom, San Diego, CA, USA). The promoter regions of the upstream sequence were chosen according to the website: http://genome.ucsc.edu. PCR primers used in this system (Table 2) were designed using predict software (Methyl Primer Express v1.0.exe).

The procedures and reaction system were reported before [15]. The region analyzed and the CpG sites of the upstream sequence are shown in Figure 2 and Table 3. The same experiments were repeated in triplicate. The methylation level was presented as the ratio of methylated cytosines over the total number of methylated and unmethylated cytosines.

### 2.6. Western Blot Analysis

Western blot was carried out as described previously [16]. Infarction border myocardial tissue samples were harvested for Western blot. Anti-ALDH2 antibody (1 : 500) was purchased from Santa Cruz Biotechnology Inc.

### 2.7. Total RNA Preparation and Real-Time PCR of ALDH2

Total RNA was extracted from the infarction border zone using reverse transcription and real-time PCR was performed according to the manufacturer's protocol of PrimeScriptTM RT reagent kit and SYBR Premix Ex TaqTM kit (TaKaRa). Real-time quantification was applied in Bio-Rad IQ5 real-time PCR machine. Relative expression of ALDH2 gene was calculated by using 2^−ΔΔCt^ method. Primers are the same as described previously [6].

### 2.8. Global Methylation Level of Myocardial Cell

Global methylation level of myocardial cell was examined with Methylated DNA quantification kit (purchased from Epigentek). All steps were followed according to the protocol of kit.

### 2.9. Inhibiting DNA Methyltransferase by Decitabine

Adult male Sprague-Dawley rats were administered intraperitoneal DNA methyltransferase (DNMT) inhibitor, Decitabine (DAC), by 1 mg/kg once a day for 7 days. Myocardial infarction model procedure was performed on half of rats intervened by DAC which is DAC+MI group, while the group with only DAC intervention is DAC group. Heart tissues of both groups were harvested seven days after myocardial infarction model procedure. Global DNA methylation level and ALDH2 upstream target sequence methylation level were examined with Methylated DNA quantification Kit and MassARRAY, respectively, and expressions of related proteins were determined with Western blot.

### 2.10. Statistical Analysis

Data were analyzed using GraphPad Prism (version 5.0; GraphPad Software Inc., San Diego, CA, US) and SPSS (version 15.0; SPSS Inc., Chicago, IL, US). ANOVA with post-hoc test was performed to compare the methylation levels between MI and Sham groups and between different MI groups.

### 2.11. Bioinformatics

The transcription factor binding elements were predicted using the known DNA-binding profiles (JASPAR, http://jaspar.genereg.net/) at the position of polymorphic sites (Primary profile similarity is up to 80%).

## 3. Results

### 3.1. Decreased ALDH2 Protein and mRNA Expression in Infarction Border Zone after MI

ALDH2 protein expression result and real-time PCR result are shown in Figures 3(a) and 3(b), respectively. ALDH2 protein and mRNA expression on infarction border zone was significantly and similarly decreased at 1 week, 2 weeks, and 3 weeks after MI (all *P* < 0.05 versus Sham).

### 3.2. DNA Methylation in ALDH2 Core Promoter Region

**Figure 1:**
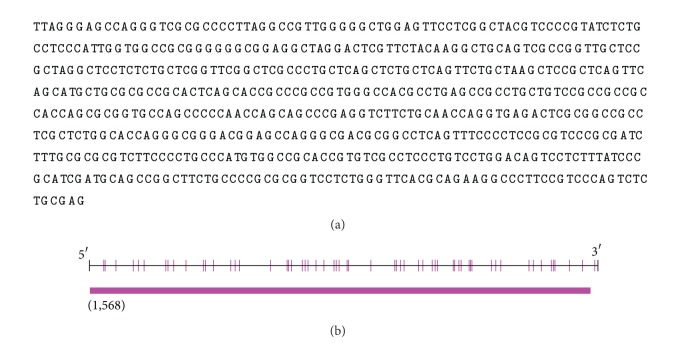
ALDH2 core promoter sequence. (a) ALDH2 core promoter sequence. (b) CpG distribution of ALDH2 core promoter was analyzed in Methyl Primer Express; red pillar stands for CpG sites and bar underlying represents CpG island (criteria: up to 200 bp, and GC percentage: up to 50%).

**Figure 2:**
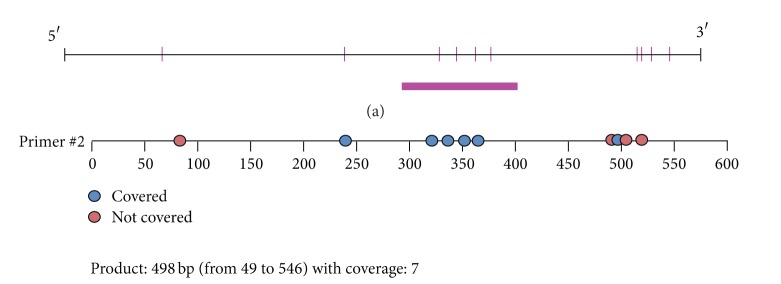
Upstream sequence of ALDH2 core promoter. (a) The chart is the analysis result of Methyl Primer Express; red pillars stand for CpG sites, and bar underlying highlights CpG high density region; (b) another analysis in the same region; blue dots represent valid CpG sites while red dots are invalid which cannot be detected in MassARRAY. Valid dots were numbered.

**Figure 3:**
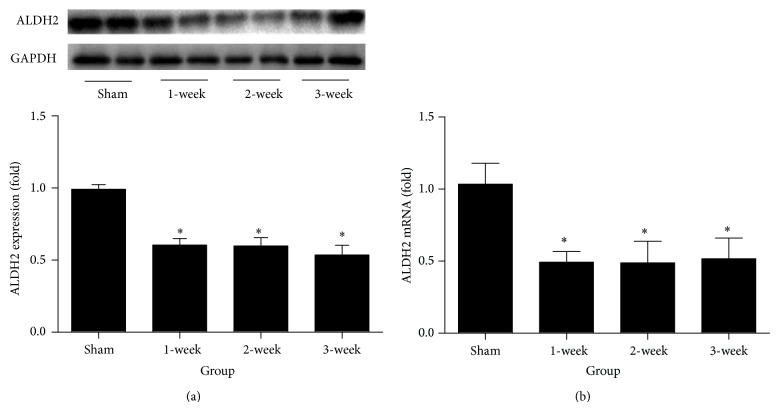
Result and analysis chart of Western blot and real-time PCR. (a) Protein level of ALDH2 was determined by Western blot. (b) mRNA of ALDH2 was determined by real-time PCR. ^*^ *P* < 0.05 versus Sham.

The sequence of CpG sites in ALDH2 core promoter was shown in Figure 1, and 58 valid CpG sites were detected in BSP (Table 3), which covered all possible CpG sites in ALDH2 core promoter region. Furthermore, this region was qualified as CpG island (criteria: up to 200 bp, and GC percentage: up to 50%). Our results showed that the baseline methylation levels of all CpG sites in ALDH2 core promoter were at level 0 (Table 4, DNA methylation level standard: level 0 is no methylation, level 1 is 1~33%, level 2 is 33%~67%, level 3 is 68%~99%, and level 4 is 100%) in all 4 examined groups, and the BPS result indicated CpG sites of rat ALDH2 core promoter were not affected by DNA methylation; therefore, MI did not trigger the alteration of DNA methylation in ALDH2 core promoter region.

### 3.3. Aberrant DNA Methylation Level in Upstream Sequence of ALDH2 Promoter after MI

To maintain the homogenous characteristic of tissue between BSP and MassARRAY, tissue samples from the same myocardial region were used to examine the methylation level of upstream sequence of ALDH2 core promoter. A 500 to 600 bp length region of DNA sequence was targeted, which existed in upstream direction of ALDH2 promoter and was 763 bp distant from rat ALDH2 core promoter. Valid CpG sites were numbered and methylation level was detected in this region with SQ2 primer.

**Figure 4:**
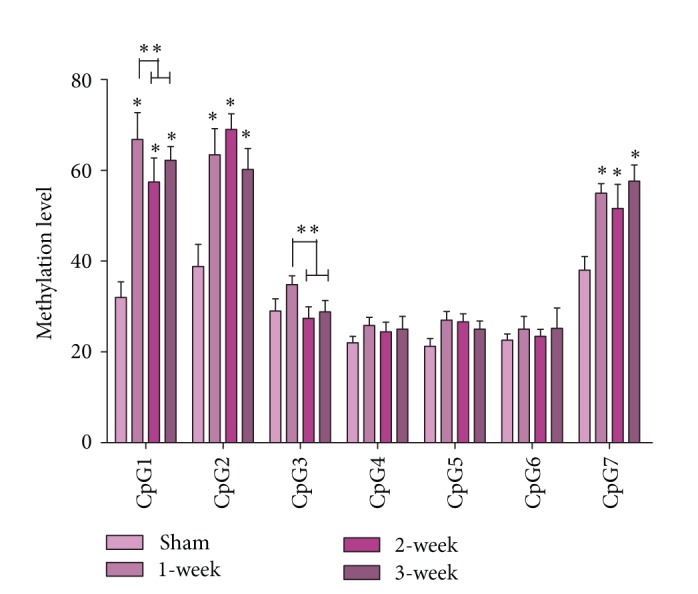
Methylation level in the upstream of ALDH2 core promoter. CpG numbers are described in Table 3. SQ2 is chosen as primer, which is described in Table 2. ^*^ *P* < 0.01 versus Sham, ^**^ *P* < 0.05 versus 1st MI group.

The mean methylation levels of seven CpG sites varied across different CpG units, ranging from 21.2% to 69.0% (Figure 4). SQ2 primer MassARRAY analysis suggested there were statistical differences in DNA methylation level between Sham group and MI groups at CpG1, CpG2, CpG3, and CpG7 (*P* < 0.01, Figure 4). No significant difference was detected between MI groups in CpG2, CpG4, CpG5, CpG6, and CpG7 (*P* > 0.1), but, compared with the other two MI groups, 1-week MI groups showed a higher DNA methylation level of at CpG 1 and CpG 3 sites (Figure 4, *P* < 0.05 versus 1 week), and no difference was detected between 2-week and 3-week MI groups (Figure 4, *P* > 0.05).

### 3.4. Verification of Methylation Level in ALDH2 Promoter Upstream Sequence

**Figure 5:**
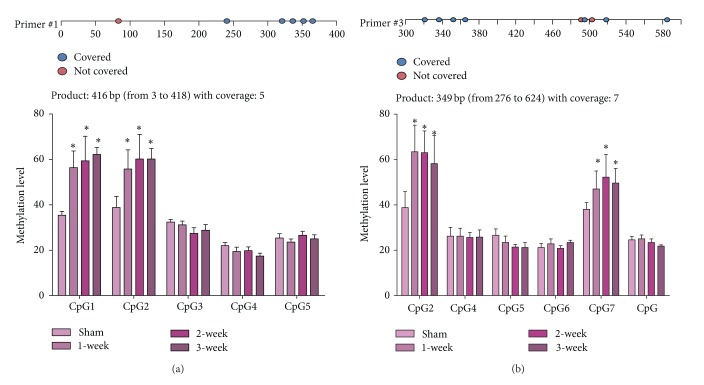
Two more pairs of primer for verification of upstream sequence. (a) SQ1 Primer and SQ1 MassARRAY result. (b) SQ3 primer MassARRAY result. Both (a) and (b) are performed according to Table 2 primer pattern, and CpG numbers are showed in Table 3. ^*^ *P* < 0.01 versus Sham.

Further MassARRAY evaluation and verification were performed with SQ1 and SQ3 primers, which contained part of valid CpG sites of SQ2 primer, respectively (Table 2). SQ1 primer analysis showed there were no significant differences among MI groups at all 5 CpG sites (Figure 5, *P* > 0.05), whereas the difference still existed at CpG1 and CpG2 between Sham and MI groups (Figure 5, *P* < 0.01 versus Sham).

SQ3 primer results were similar among MI groups (Figure 5, *P* > 0.05). CpG2 and CpG7 methylation level differences were still maintained as shown in SQ2 and SQ1 (Figure 5, *P* < 0.01 versus Sham). No difference of methylation level was found at CpG4 and CpG5 in SQ3 (Figure 5, *P* > 0.05 versus Sham).

Taken together, hypermethylation was evidenced at CpG1, CpG2, and CpG7 in infarction border zone. It indicated that SOX10 (SRY-related HMG-box 10, SRY for sex determining region Y) was the most possible TF binding to target sequence (Table 5).

### 3.5. Global Methylation Level Was Decreased by DAC

**Figure 6:**
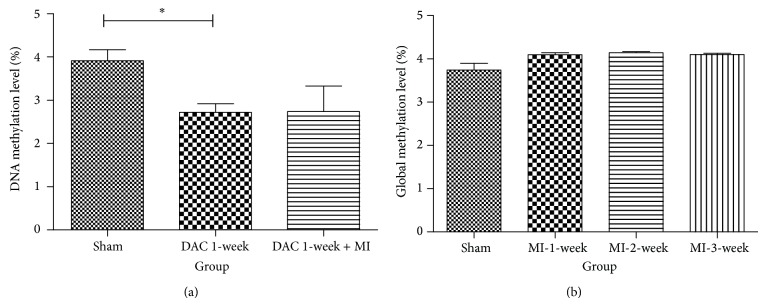
Global methylation level of myocardial cell. (a) Global methylation level of myocardial cell decreased after the intervention of DAC (^*^ *P* < 0.05). (b) No significant difference of DNA methylation level was detected between Sham and MI groups.

DNA global methylation level of myocardial cells from the infarction border was significantly reduced after intervention of DAC (Figure 6(a), *P* < 0.05) while, in the groups without DAC intervention, no significant difference of DNA methylation level was detected between Sham and MI groups (Figure 6(b)).

### 3.6. DAC Intervention Reduced DNA Methylation Level of Target Upstream Sequence and Related Proteins Expression

MassARRAY analysis was performed in target upstream sequence with primer SQ1 for DAC group and DAC+MI group. Western blot of related proteins, including DNMT1 and ALDH2, was performed.

**Figure 7:**
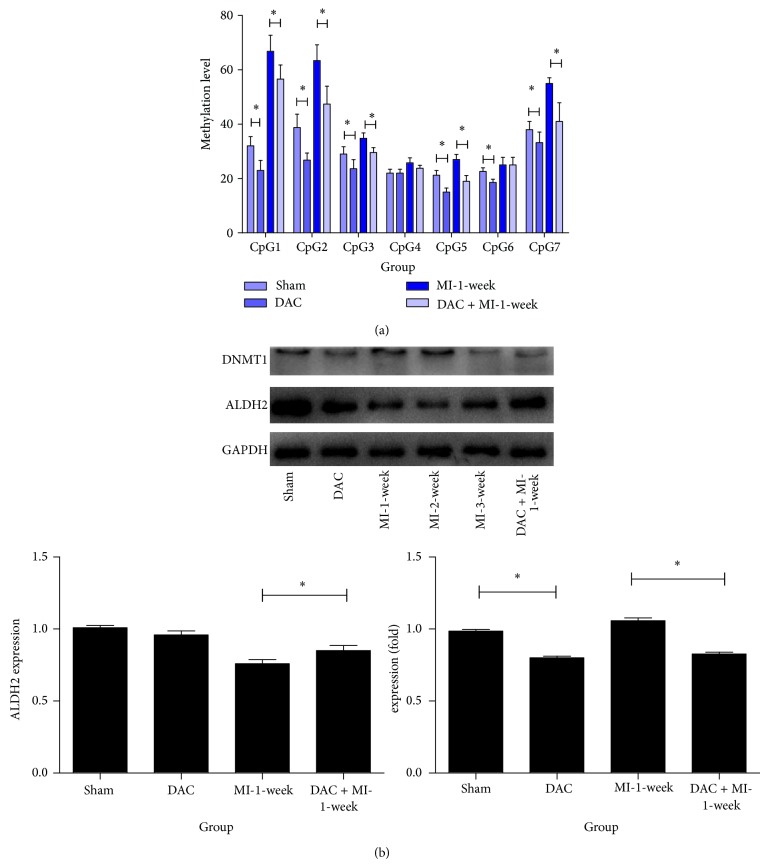
DNA methylation level of target upstream sequence and related proteins expression. (a) MassARRAY analysis result of target upstream sequence for DAC group and DAC + MI group. Sham group and MI-1-week group are chosen as comparative groups (^*^ *P* < 0.05). (b) Western blot of related proteins, including DNMT1 and ALDH2 (^*^ *P* < 0.05).

DAC intervention reversed hypermethylation of target upstream sequence in MI (Figure 7(a), *P* < 0.05). Furthermore, DAC inversed the reduction of ALDH2 after MI while it had no significant effect on baseline ALDH2 expression (Figure 7(b), *P* < 0.05).

## 4. Discussion

Our study demonstrates that DNA methylation might contribute to the upstream regulation of ALDH2 after myocardial infarction, suggesting that there is an association between ALDH2 promoter hypermethylation and myocardial infarction (The location of ALDH2 in* Rattus norvegicus* is 12q16. The RefSeq number is NC_005111.4. All information of target gene is referred from the database of Pubmed GenBank). Additionally, we combined two examination methods of DNA methylation, which might offer a more concrete result and contribute to a thorough understanding for ALDH2 promoter region with an ideal cost-effective outcome.

It has been substantially shown that DNA methylation plays a pivotal role in the regulation of DNA transcription level [9], and DNA methylation is one of the most important epigenetic changes [7]. The effects of DNA methylation upon transcription regulation are multiple [10, 12], and the change of DNA methylation level in a specific region, mostly occurring at CpG sites or CpG island [17–19], is critically related to the final results of DNA methylation upon transcription regulation [20, 21].

We hypothesized that DNA methylation may actively participate in the pathogenesis of myocardial infarction and is related to ALDH2 decrease after MI. Till now, there is no report exploring the association between ALDH2 gene methylation and myocardial infarction. In this study, DNA methylation was evaluated in the present study with BSP and MassARRAY. Core promoter sequence of rat ALDH2 gene was analyzed and possible CpG sites were mapped in BSP examination. BSP analysis showed that DNA methylation did not affect ALDH2 core promoter in the setting of myocardial infarction. To overcome the limitation of BSP technique [22], MassARRAY was performed for DNA methylation examination in upstream sequence of ALDH2 promoter. MassARRAY analysis results showed there were no significant differences among MI groups at all 5 CpG sites (*P* > 0.05), whereas the difference between Sham and MI groups was found at CpG1, CpG2, and CpG7 sites (*P* < 0.01 versus Sham).

Our first MassARRAY experiment with SQ2 suggested there were slightly significant differences in MI groups (Figure 4), while subsequent experiments with SQ1 and SQ3 were carried out to test the accuracy of SQ1 result, and results showed there were no differences in MI groups. Previous methodology research also suggested confounding factors could influence the accuracy of MassARRAY [23], so verification issue is essential and should be addressed in MassARRAY.

In our study, BSP was performed to examine DNA methylation level of ALDH2 core promoter, which turned out to be a negative result. However, MassARRAY was performed in this region as well, while the preliminary PCR result of MassARRAY was invalid because of extensively high production of dimers, and regulation of PCR temperature or consequences of primers had no effect on eliminating dimers, so MassARRAY final result is far from ideal. It is suggested that the appendix of Primer is combined with specific DNA sequence in ALDH2 core promoter to trigger high production of dimer, while appendix of Primer is constant and essential for mass spectrum in MassARRAY, and ALDH2 core promoter cannot be changed. The preferential method was supposed to choose BSP instead of MassARRAY, and this result also indicated the limitation of MassARRAY in some specific genes; moreover, combination of MassARRAY and BSP may be a rational procedure for detecting DNA methylation level of specific genes, especially in ALDH2 promoter of* Rattus norvegicus*.

Global methylation status in this study was detected to get a comprehensive evaluation of myocardial DNA methylation level. Though study in cancers suggested that hypomethylation of ALDH2 belongs to part of a global DNA methylation change [24], our study indicated there was just nonsignificant increase in global DNA methylation of myocardial cells after MI (Figure 6(b)), while upstream sequence of ALDH2 core promoter was hypermethylated. The possible explanation for this result is the fact that global DNA methylation is typically an integration of all CpG sites, in which significant changes of specific sequences can be neutralized, and chip screening might be the preferential method for analyzing such issues [25].

Further experiment was carried out to determine the potential mechanism for hypermethylation. Target sequence, which is upstream sequence of ALDH2 core promoter, possesses a baseline methylation status, and DNMT 1 can influence the maintenance of DNA methylation [26], so we targeted DNMT1 as a potential enzyme responsible for the change of target sequence. Our result suggested DAC intervention indeed deceased DNA methylation status, both in global methylation (Figure 6(a)) and in specific CpG sites in target sequence (Figure 7(a)), and DAC intervention upregulated ALDH2 expression via inhibiting DNMT1 (Figure 7(b)). All these indicated that DNMT1 increased the methylation level of target sequence after MI and, hence, reduced ALDH2 expression. On the other hand, DAC intervention reduced the global methylation level (Figure 6(a)), which is consistent with cancer studies [27].

**Figure 8:**
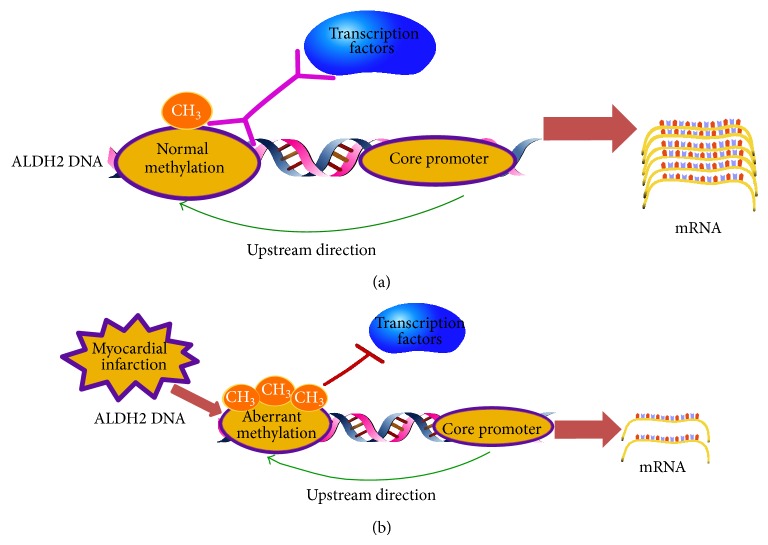
Potential mechanism for alternation of DNA methylation level in ALDH2 core promoter upstream sequence.

The underlying mechanism for the above change could be that hypoxia induced the increase of DNMT1, at least in its enzyme expression, and that DNMT1 subsequently enhanced DNA methylation status of target sequence; finally, ALDH2 was downregulated (Figure 8). One potential link between hypoxia and DNMT1 could be HIF-1*α*, which is suggested as a possible pathway for the alteration of DNMT and explored in a previous study [28]. Moreover, bioinformatics analysis suggested SOX10 could be a potential TF binding with target sequence (Table 5); further researches are warranted to verify the above hypothesis.

Besides methylation changes of ALDH2 after myocardial infarction, there might be a series of genes, which would also face methylation changes in the setting of MI. Breitling et al. reported that F2RL3 gene hypermethylation was associated with the incidence of MI [29]. There was also the report stating that DNA methylation at GNASAS gene was modestly higher in MI patients [30]. Chang et al. found that there was hypermethylation in FGF2 gene promoter in MI patients [31]. Epidemic research demonstrated that global DNA methylation was significantly elevated in male MI patients [32], and Fiorito et al. showed three differentially methylated regions (TCN2 promoter, CBS 5′UTR, and AMT gene-body) in male MI patients [33].

As a key enzyme against myocardial ischemia injury, ALDH2 is also regulated under some other potential mechanisms which could induce the declination of its expression or enzyme activity. In our previous study, ALDH2 was proved to protect myocardial cells against ischemia-reperfusion injury through regulation of autophagy via AMPK- and Akt-mTOR signaling [3]; furthermore, microRNA-34a was shown to reduce the expression of ALDH2 via binding on ALDH2 mRNA in MI rats [6]. Lagranha et al. found that PKC could induce declination of ALDH2 phosphorylation and attenuate ALDH2 both in vivo and in isolated rat heart model of myocardial ischemia reperfusion [34]. Yu et al. demonstrated PI3K/Akt-dependent signaling pathway was associated with the decrease of ALDH2 in MI rats [35]. It is reported SIRT3-mediated deacetylation decreased ALDH2 activity [36].

There are still some limitations in our study. We only observed that MI was associated with aberrant hypermethylation of CpG sites; future studies are needed to prove the mechanism how myocardial ischemia injury could affect DNA methylation level in target sequence. This procedure may involve specific gene, such as NKCC1 gene as reported [37], or alteration of other DNMTs, such as DNMT3a or DNMT3b, which is a key enzyme family for de novo DNA methylation progress [27, 38] and reported involved after MI [39].

## 5. Conclusion

In summary, the present study suggests DNA methylation has an effect on the upstream sequence of ALDH2 promoter, which is possibly associated with the decrease of ALDH2 expression after myocardial infarction. These findings could be a potential epigenetic explanation for ALDH2 decrease after myocardial infarction or ischemia injury.

## Figures and Tables

**Table 1:** Primer sequences, product length, and CpG units used for BSP.

Primer number	Forward primer (5′-3′)	Reverse primer (5′-3′)	Product length (bp)	CpG unit
M1	GTGAGTTGGGTAGGGATGGA	CRTCTTCCCCTACCCATATAACC	101	9
M2	GGGATAAAGAGGATTGTTTAGGATA	ATACCAACCCCCAACCAAC	139	19
M3	GTTGGTTGGGGGTTGGTAT	CCRTATCTCTACCTCCCATTAATAACC	177	20
M4	GAGTAATYGGYGATTGTAGTTTTGTAGA	ATCCCCATATTCTACAAACTCCATCTC	105	10

**Table 2:** Primer sequences, product length, and CpG units used for MassARRAY.

Primer number	Forward primer (5′-3′)^1^	Reverse primer (5′-3′)^2^	Product length (bp)	CpG unit
SQ1	TTAAGGATTTGTTTGTATTTAATTGG	CAAACAATACCACAATTTATATCTTTTA	416	6
SQ2	AGATTTGGAGAGATGGTTTAGTGGT	ACCCATTTCCTACAAAAAAATATCC	498	10
SQ3	TTAAAAGATATAAATTGGGGTTGGG	ACCAACAAAACCCCTCTAAATAAAC	349	9

^1^For 10-mer tag, CAGTAATACGACTCACTATAGGGAGAAGG was added and ^2^for T7 promoter tag AGGAAGAGAG was added.

**Table 3:** Upstream sequence of ALDH2 core promoter in detail.

	AGACCTGGAGAGATGGCTCAGTGGTTAAGAGCAC**CG**ACTGCTCTTCCAGAGGTCCTGAGTTCAAATCCCAG	
	0	
	CAGGTTCACAACCATTTGTAATGAGATCTGATGCCCTCCTCTGGTATGTCTGAAGACAGCTACAGTGTACTTAT	
		
	ATATAACAATAAATAAAATTAAAAAAAAAAAAAGCAGTAGACATCC**CG**TGAGTTAGTGGCTCTTCCTCTGTGA	
	1	
	ATTAGTAAGTCAAAAGACACAAACTGGGGTTGGGGATTTAGCTCAGTGGTAGAG**CG**CTTGCCTAGGAAG**C**	
	2 3	
	**G**CAAGGCCCTGGGTT**CG**GTCCCCAGCTC**CG**GGAAAAAAAAAAAAAAAAAAGAACCAAAAGACACAAACT	
	4 5	
	GTGGCATTGTTTGTAATCACACAAGATTGGAAGCCACCTTAAATGTCACCCACAGAACTTTCTTTAAATGGG	
		
	AATGCACAGGTAG*CG*AA**CG**AAACAG*CG*AGTAAACCAGTGAA**CG**GGGACACCTTCCTGCAGGAAATGGGT	
	6 7	

The valid CpG sites are written in bold, while invalid CpG sites are written in italic. Valid CpG sites are numbered.

**Table:** (a) M1

CpG number	1	2	3	4	5	6	7	8	9
A1	0	0	0	0	0	0	0	0	0
A2	0	0	0	0	0	0	0	0	0
A3	0	0	0	0	0	0	0	0	0
A4	0	0	0	0	0	0	0	0	0
A5	0	0	0	0	0	0	0	0	0
B1	0	0	0	0	0	0	0	0	0
B2	0	0	0	0	0	0	0	0	0
B3	0	0	0	0	0	0	0	0	0
B4	0	0	0	0	0	0	0	0	0
B5	0	0	0	0	0	0	0	0	0
C1	0	0	0	0	0	0	0	0	0
C2	0	0	0	0	0	0	0	0	0
C3	0	0	0	0	0	0	0	0	0
C4	0	0	0	0	0	0	0	0	0
C5	0	0	0	0	0	0	0	0	0
Sh1	0	0	0	0	0	0	0	0	0
Sh2	0	0	0	0	0	0	0	0	0
Sh3	0	0	0	0	0	0	0	0	0
Sh4	0	0	0	0	0	0	0	0	0
Sh5	0	0	0	0	0	0	0	0	0

**Table:** (b) M2

CpG number	1	2	3	4	5	6	7	8	9	10	11	12	13	14	15	16	17	18	19
A1	0	0	0	0	0	0	0	0	0	0	0	0	0	0	0	0	0	0	0
A2	0	0	0	0	0	0	0	0	0	0	0	0	0	0	0	0	0	0	0
A3	0	0	0	0	0	0	0	0	0	0	0	0	0	0	0	0	0	0	0
A4	0	0	0	0	0	0	0	0	0	0	0	0	0	0	0	0	0	0	0
A5	0	0	0	0	0	0	0	0	0	0	0	0	0	0	0	0	0	0	0
B1	0	0	0	0	0	0	0	0	0	0	0	0	0	0	0	0	0	0	0
B2	0	0	0	0	0	0	0	0	0	0	0	0	0	0	0	0	0	0	0
B3	0	0	0	0	0	0	0	0	0	0	0	0	0	0	0	0	0	0	0
B4	0	0	0	0	0	0	0	0	0	0	0	0	0	0	0	0	0	0	0
B5	0	0	0	0	0	0	0	0	0	0	0	0	0	0	0	0	0	0	0
C1	0	0	0	0	0	0	0	0	0	0	0	0	0	0	0	0	0	0	0
C2	0	0	0	0	0	0	0	0	0	0	0	0	0	0	0	0	0	0	0
C3	0	0	0	0	0	0	0	0	0	0	0	0	0	0	0	0	0	0	0
C4	0	0	0	0	0	0	0	0	0	0	0	0	0	0	0	0	0	0	0
C5	0	0	0	0	0	0	0	0	0	0	0	0	0	0	0	0	0	0	0
Sh1	0	0	0	0	0	0	0	0	0	0	0	0	0	0	0	0	0	0	0
Sh2	0	0	0	0	0	0	0	0	0	0	0	0	0	0	0	0	0	0	0
Sh3	0	0	0	0	0	0	0	0	0	0	0	0	0	0	0	0	0	0	0
Sh4	0	0	0	0	0	0	0	0	0	0	0	0	0	0	0	0	0	0	0
Sh5	0	0	0	0	0	0	0	0	0	0	0	0	0	0	0	0	0	0	0

**Table:** (c) M3

CpG number	1	2	3	4	5	6	7	8	9	10	11	12	13	14	15	16	17	18	19	20
A1	0	0	0	0	0	0	0	0	0	0	0	0	0	0	0	0	0	0	0	0
A2	0	0	0	0	0	0	0	0	0	0	0	0	0	0	0	0	0	0	0	0
A3	0	0	0	0	0	0	0	0	0	0	0	0	0	0	0	0	0	0	0	0
A4	0	0	0	0	0	0	0	0	0	0	0	0	0	0	0	0	0	0	0	0
A5	0	0	0	0	0	0	0	0	0	0	0	0	0	0	0	0	0	0	0	0
B1	0	0	0	0	0	0	0	0	0	0	0	0	0	0	0	0	0	0	0	0
B2	0	0	0	0	0	0	0	0	0	0	0	0	0	0	0	0	0	0	0	0
B3	0	0	0	0	0	0	0	0	0	0	0	0	0	0	0	0	0	0	0	0
B4	0	0	0	0	0	0	0	0	0	0	0	0	0	0	0	0	0	0	0	0
B5	0	0	0	0	0	0	0	0	0	0	0	0	0	0	0	0	0	0	0	0
C1	0	0	0	0	0	0	0	0	0	0	0	0	0	0	0	0	0	0	0	0
C2	0	0	0	0	0	0	0	0	0	0	0	0	0	0	0	0	0	0	0	0
C3	0	0	0	0	0	0	0	0	0	0	0	0	0	0	0	0	0	0	0	0
C4	0	0	0	0	0	0	0	0	0	0	0	0	0	0	0	0	0	0	0	0
C5	0	0	0	0	0	0	0	0	0	0	0	0	0	0	0	0	0	0	0	0
Sh1	0	0	0	0	0	0	0	0	0	0	0	0	0	0	0	0	0	0	0	0
Sh2	0	0	0	0	0	0	0	0	0	0	0	0	0	0	0	0	0	0	0	0
Sh3	0	0	0	0	0	0	0	0	0	0	0	0	0	0	0	0	0	0	0	0
Sh4	0	0	0	0	0	0	0	0	0	0	0	0	0	0	0	0	0	0	0	0
Sh5	0	0	0	0	0	0	0	0	0	0	0	0	0	0	0	0	0	0	0	0

**Table:** (d) M4

CpG number	1	2	3	4	5	6	7	8	9	10
A1	0	0	0	0	0	0	0	0	0	0
A2	0	0	0	0	0	0	0	0	0	0
A3	0	0	0	0	0	0	0	0	0	0
A4	0	0	0	0	0	0	0	0	0	0
A5	0	0	0	0	0	0	0	0	0	0
B1	0	0	0	0	0	0	0	0	0	0
B2	0	0	0	0	0	0	0	0	0	0
B3	0	0	0	0	0	0	0	0	0	0
B4	0	0	0	0	0	0	0	0	0	0
B5	0	0	0	0	0	0	0	0	0	0
C1	0	0	0	0	0	0	0	0	0	0
C2	0	0	0	0	0	0	0	0	0	0
C3	0	0	0	0	0	0	0	0	0	0
C4	0	0	0	0	0	0	0	0	0	0
C5	0	0	0	0	0	0	0	0	0	0
Sh1	0	0	0	0	0	0	0	0	0	0
Sh2	0	0	0	0	0	0	0	0	0	0
Sh3	0	0	0	0	0	0	0	0	0	0
Sh4	0	0	0	0	0	0	0	0	0	0
Sh5	0	0	0	0	0	0	0	0	0	0

(a), (b), (c), and (d) represent four pairs of BSP primers, respectively, which were numbered in Table 1.

(A): for 1st week MI group.

(B): for 2nd week MI group.

(C): for 3rd week MI group and SH for Sham group.

**Table 5:** Predicted transcription factors by JASPAR database.

Model ID	Model name	Score	Relative score	Start	End	Strand	Predicted site sequence
MA0152.1	NFATC2	6.724	0.829025039347648	26	32	1	TCTTCCT
MA0442.1	SOX10	5.846	0.863982829885209	31	36	1	CTCTGT
MA0099.2	JUN::FOS	5.636	0.816470638357913	36	42	1	TGAATTA
MA0040.1	Foxq1	11.209	0.889149675048185	182	192	1	CATTGTTTGTA
MA0442.1	SOX10	8.625	0.987356608195436	182	187	1	CATTGT
MA0442.1	SOX10	4.805	0.817767607423551	186	191	1	GTTTGT
MA0038.1	Gfi1	7.464	0.846275818186275	190	199	1	GTAATCACAC
MA0116.1	Zfp423	6.657	0.801580348722667	210	224	1	GCCACCTTAAATGTC
MA0160.1	NR4A2	8.782	0.899753978671343	219	226	1	AATGTCAC
MA0442.1	SOX10	6.636	0.899054900725468	235	240	1	CTTTCT
MA0038.1	Gfi1	6.129	0.806171416304315	279	288	1	TAAACCAGTG

Putative sites were predicted with the setting of ALDH2 promoter upstream sequence, and predicted scores were ⩾80%.
